# Editorial for the Special Issue on Advances in Ultra-Precision Machining Technology and Applications

**DOI:** 10.3390/mi13122093

**Published:** 2022-11-28

**Authors:** Benny C. F. Cheung, Jiang Guo

**Affiliations:** 1 State Key Laboratory of Ultra-Precision Machining Technology, Department of Industrial and Systems Engineering, The Hong Kong Polytechnic University, Kowloon, Hong Kong; 2 School of Mechanical Engineering, Dalian University of Technology, Dalian 116024, China

Ultra-precision machining technology has been widely used in the manufacture of many mission-critical components for various industrial areas, such as the advanced optics, photonics aerospace, automotive, telecommunications, biomedical and energy and environmental sectors, among others. The increasing degree of geometrical complexity, requirement of high precision and the evolution of the materials used for machined workpieces have led to many research challenges in different fields, including ultra-precision machining technologies, novel machining processes, cutting mechanics, surface generation mechanisms, novel machine design, advanced sensing, machine metrology, accurate control of the machining process through modeling and simulation of ultra-precision machining processes, error compensation, materials sciences, measurement and on-machine metrology, as well as advanced applications for functional uses.

This Special Issue offers a high-quality collection of 17 papers detailing the latest research results and findings in the field of ultra-precision machining technology and related applications. These papers cover various aspects of this topic, including multi-physical coupling simulation of electrochemical machining and aerostatic spindle, error measurement and compensation, femtosecond-laser-assisted etching, numerical modeling of cutting force and stress, prediction of milling stability, influence of tribological characteristics, tribochemical mechanical polishing, low-pressure lapping, thermal effect, micromachining innovation design, single-particle erosion mechanism, dynamic performance of aerostatic thrust bearing, fly-cutting process and ultrasonic-vibration-assisted cutting.

Highlights of this collection of papers are as follows. Li et al. [1] present a study focused on the forming mechanism of cooling hole electrolytic machining using multi-physical field coupled simulation and experimental observation. An investigation of the main error sources was conducted by Xiang et al. [2] for the error motion measurement of a precision shafting based on a T-type capacitive sensor. Wang et al. [3] report a femtosecond-laser-assisted dry-etching technology that can be utilized to realize the fabrication of silicon microlenses. A new cutting force coefficient model is established Li et al. [4], revealing the influence of the cutting-edge radius on the cutting process. An updated full-discretization method is presented by Ma et al. [5] for milling stability prediction based on cubic spline interpolation. Nagîț et al. [6] elaborate the tribological behavior of test piece surfaces, analyzing the changes in the values of the coefficient of friction and loss of mass that appear over time. Qi et al. [7] demonstrate the mechanism underlying oxygen production and the tribochemical reaction mechanisms of SiC during fixed abrasive tribochemical mechanical polishing. Yu et al. [8] describe the modeling and simulation of the surface generation mechanism of a novel low-pressure lapping method using the finite element method, indicating that rotational speed plays a major role in this process. A three-degrees-of-freedom (3-DOF) quasi-static kinematics model is established by Lei et al. [9] for motion errors containing the thermal effect for the hydrostatic guideway. Experimental results show that the model has a certain effect on thermal error prediction. Wang et al. [10] present a knowledge-based holistic framework that enables process planners to achieve micromachining innovation design by analyzing innovation design procedures and available knowledge sources. Cao et al. [11] combine smoothed particle hydrodynamics (SPH) simulation and an experiment to investigate the single-particle erosion mechanism of optical glass and verify the effect of impact velocity and particle size on material removal rate. Wang et al. [12] utilize the FLUENT software to simulate and analyze the impact of throttling characteristics of small orifices on the stiffness and stability optimization of aerostatic thrust bearings. Dai et al. [13] analyze the electrochemical machining (ECM) process in a film-cooling hole by conducting a multi-physics coupling simulation on the basis of Faraday’s law and a fluid heat transfer mathematical model. An et al. [14] utilize simulation to explore the causes of potassium dihydrogen phosphate (KDP) chip formation in the single-point diamond fly-cutting process, and micro-cracks on the machined surface are analyzed based on thermo-mechanical coupling and chip morphology. Guan et al. [15] propose a high-precision machining method for weak-stiffness mirrors based on the fast tool servo system and realize a clamping error with a peak-to-valley (PV) value of 5.2 µm and a cutting error with a PV value of 1.6 µm. Zhang et al. [16] demonstrate a 104 kHz ultrasonic-vibration-assisted cutting system could achieve a constant surface roughness of about 3 nm to 4 nm in machining steel optical modules with 0–15° slope degrees. Finally, Chen et al. [17] establish a multi-field coupling 5-DOF dynamics model for the aerostatic spindle considering the interaction between the air film, spindle shaft and the motor.
